# Children’s inhibition skills are associated with their P3a latency—results from an exploratory study

**DOI:** 10.1186/s12993-022-00202-7

**Published:** 2022-12-01

**Authors:** Tanja Linnavalli, Outi Lahti, Minna Törmänen, Mari Tervaniemi, Benjamin Ultan Cowley

**Affiliations:** 1 Cicero Learning, Faculty of Educational Sciences, University of Helsinki, Helsinki, Finland; 2 Cognitive Brain Research Unit, Faculty of Medicine, University of Helsinki, Helsinki, Finland; 3 Department of Education, Faculty of Educational Sciences, University of Helsinki, Helsinki, Finland; 4 Department of Psychology and Logopedics, Faculty of Medicine, University of Helsinki, Helsinki, Finland; 5 Institute for Professionalization and System Development, University of Teacher Education in Special Needs, Zurich, Switzerland; 6 Cognitive Science, Department of Digital Humanities, Faculty of Arts, University of Helsinki, Helsinki, Finland

**Keywords:** P3a, Children, Inhibition, Set-shifting, Modified flanker task

## Abstract

**Background:**

The P3a response is thought to reflect involuntary orienting to an unexpected stimulus and has been connected with set-shifting and inhibition in some studies. In our exploratory study, we investigated if the amplitude and the latency of the P3a response were associated with the performance in a modified flanker task measuring inhibition and set-shifting in 10-year-old children (N = 42). Children participated in electroencephalography (EEG) measurement with an auditory multifeature paradigm including standard, deviating, and novel sounds. In addition, they performed a separate flanker task requiring inhibition and set-shifting skills.

**Results:**

The P3a latencies for deviant sounds were associated with the reaction time reflecting inhibition: the shorter the response latencies were, the faster the reaction time was. The P3a latencies for novel sounds were not linked to the reaction times reflecting either inhibition or set-shifting. In addition, the magnitude of the P3a response was not associated with the performance in the flanker task.

**Conclusions:**

Our results suggest that P3a response latency and reaction speed reflecting inhibitory skills are based on shared neural mechanism. Thus, the present study brings new insight to the field investigating the associations between behavior and its neural indices.

## Background

The P3a response of the event-related potentials (ERPs) has been proposed to directly index an epistemic (uncertainty-reducing) stage of attention allocation [1], and it appears to have generators in the frontal cortex [2–7]. Executive functions seem also to rely on the frontal brain regions and their multifold connections with other brain areas [8, 9]. According to some studies, the P3a appears to be associated with set-shifting and inhibition of executive functions [10–13], and e.g., Barceló et al. [11, 14] have suggested a common neural network appearing behind both P3a generation and set-shifting. Furthermore, the maturation of prefrontal areas seems to precede the development of executive functions [15, 16]. Thus, this maturation might contribute to concurrent changes in the P3a response and the performance in tests measuring executive functions. However, empirical literature on this relationship in childhood is scarce, suggesting that we need more studies on the role of P3a in executive functions (EF) during childhood. In the current exploratory study, we report cross-sectional data collected from school-aged children using measurements for inhibition and set-shifting (modified flanker) task and auditory P3a recordings with multifeature paradigm.

### Inhibition and set-shifting

Inhibition and set-shifting skills are functions that are categorized under the umbrella term of executive functions (EF), a set of higher order cognitive processes that enable and regulate goal-oriented actions and are thus crucial for acting in a meaningful way in life. There is no clear consensus about the exact subfunctions that are included in the concept of EF, but according to an influential model by Miyake et al. [17], executive functions are comprised of three partially overlapping but differentiable abilities that are: inhibition of automatic reactions, set-shifting (or shifting), and working memory (referred to as “information updating and monitoring” in Miyake et al. [17]). Although these functions operate together and are difficult to disentangle from each other, investigating them separately may bring knowledge of their developmental trajectories and differential contribution they give to behavior [18].

Inhibition refers to an ability to inhibit an automatic, learned, or dominant reaction [17]. This ability requires control over one’s own behavior, thoughts, and emotions to choose an appropriate reaction in a given context and it is thus crucial for regulating behavior and controlling one’s emotions. Without the capacity for inhibition, we would be at the mercy of random environmental stimuli, our own internal impulses and learned reactions. The literature acknowledges several subcategories and classifications of inhibition. However, the most important distinction seems to be between the ability to prevent a practiced, automatic motor response and the ability to ignore information that is interfering with the task at hand [see e.g. 19]. The former, response inhibition, has been called e.g., motor inhibition [20], response inhibition [21], prepotent response inhibition [22, 23] and behavioral inhibition [24]. The latter is typically referred to as e.g., conflict resolution [25, 26], resistance to distractor interference [23], and interference control [21, 24]. The present study focuses on cognitive interference control, here named as inhibition. It is measured with tests such as the Stroop test [27], anti-saccade tests [e.g. 28], and flanker tasks [29], in which both accuracy and more typically, reaction time (RT) serve as outcome measures.

Set-shifting (SS) refers to an ability to adapt one’s thinking and actions to changing situations or rules. It can be defined as a part of or overlapping with the wider concept of cognitive flexibility that is needed in real-life situations, such as when problem-solving requires a new perspective or a situation demands application of a different mindset [13]. Set-shifting ability is typically measured as the speed and accuracy with which an individual absorbs new rules in a test where the rules alternate between separate trials. Some typical tasks in measuring set-shifting capacity are e.g., Plus-minus tasks [e.g., 30, 31], Number-letter tasks [e.g., 32], Wisconsin Card Sorting task [33], or flanker task [29].

Executive functions mature throughout the childhood [34–37] and this maturation appears to be associated with the development of brain structure and processes [38–42]. EF skills may already be assessed in preschool children [see e.g., 36, 43–47], although it seems that in such small children the three functions (working memory, inhibition, and set-shifting) are not differentiable but tend to load on a single factor, rather than on three separate factors [48, 49]. It has been suggested that the three EF units follow differential developmental trajectories and may be separable from one another only in later childhood or adolescence [18, 50–53]. However, inhibition [see 36, 54–57] and set-shifting [57–60] have been measured separately in school-aged children, and have been concluded in these studies to reach adult levels around 10 to 12 years of age.

### P3a component

Auditory P3a response is a fronto-centrally maximal positive component elicited by infrequent, unpredictable stimuli in a stream of repeating sounds and peaking between 200 and 400 ms from the stimulus onset [61]. It originates from several regions of the brain, and studies have found involvement of the frontal areas [2–7] and auditory cortex [62, 63], or even the anterior cingulate gyrus [64], hippocampus [3] and parahippocampal gyri [65, for reviews, see 66, 67].

The unpredictable stimuli eliciting P3a may either be “deviants”, deviating from standard stimulus in some sound feature, such as frequency or intensity, or highly salient “novels”, sounds not sharing any resemblance with standard stimulus. In his review paper, Polich [13] proposed that P3a initiates an early attention process with an orienting change to a frontal working memory representation; this attention-driven stimulus signal then passes to temporal-parietal structures to create the P3b. The P3a and P3b are supramodal, and their amplitudes have been shown to be modulated by several exogenous and endogenous factors, such as conditions of attention orientation in auditory protocols using the oddball paradigm [64, 68, 69] and the cognitive complexity of the task [14]. Conditions of attention orientation are typically studied with active and passive auditory paradigms, in which the earlier P3a and the later P3b component may both be elicited [e.g., 69, 70]. When P3a is studied in passive paradigms, the stimuli (standards, deviants, or novels) are not actively detected and indicated, e.g., by a button push [61, 71]. Thus, by focusing on P3a it is possible to investigate the brain responses related to involuntary attention, unlike in paradigms requiring voluntary attention, in which motivation and resilience to keep attentional focus in the task may affect the responses, especially with children. As it seems that novels i.e., very salient sounds differing greatly from the standard sound in a sound stream elicit larger P3a amplitudes than deviants, i.e., smaller changes [68, 72–74], it is commonly proposed that the P3a reflects an involuntary switch of attention towards the sound that differs from the standard stimuli [13, 66, 75–77]. Thus, the larger the acoustical difference between the standard and the non-standard sound (deviant or novel) is, the larger the P3a.

Even infants and toddlers show a positive component resembling the adult-like P3a [78–82]. There is some controversy whether the P3a for small sound changes (deviants) and novel sounds is the same despite the similar frontal topography seen in both contexts [see, e.g., 13]. Supporting the view for two separate types of P3a, it seems that there are two lines of developmental trajectory for P3a magnitude, depending on the quality of the stimuli. According to some longitudinal [83–85] and cross-sectional [10, 86] studies, it appears that the magnitude increases, or matures towards adult-like positivity for small deviations in an auditory sound stream in childhood [83, 84], continuing into late adolescence [10, 85, 86]. Although some cross-sectional studies have shown contradicting results (decrease [68, 87] or no change [88, 89] in P3a magnitude with age), evidence from longitudinal studies seems to suggest that with small deviations in e.g., frequencies or phonemes, the threshold for discrimination decreases with age, possibly reflecting more efficient auditory detection. Instead, according to several cross-sectional comparisons, the P3a amplitude for salient distractors, such as completely novel sounds in an auditory stream, decreases with age [90, 91, see figures in 92], and this decrease may continue possibly even until the late adolescence [89]. Similarly, as with the P3a for deviants, some studies on the novelty P3a have found no difference between the age groups [93–95] or even results pointing to the opposite direction [96]. However, as the P3a has been shown to reflect the magnitude of distraction [64, 68, 72, 74], this decrease with age appears to be in line with the notion that children seem to be more easily distracted than adults [see e.g. 68, 90]. In other words, maturation of novel sound processing might mean more effective suppression of the involuntary attention to distracting sounds. However, it should be noted that also other phenomena, such as more effective general processing of the distractor sound or other changes in the development of general cognitive system may also lie behind this effect.

The latency of the P3a response appears to be longer in children than in adults [97], although most of the studies addressing latencies of the P3 responses tend to concentrate on the P3b responses [13]. However, some cross-sectional comparisons of different age groups have found a decrease in P3a latency with age for both novel sounds [98] and smaller sound changes [87, 99] during childhood and adolescence, suggesting that the shorter latency reflects more mature processing of deviating sounds (for a review, see [100]).

### The P3a component and behavioral measures

The P3a response has been connected with set-shifting and inhibition in some studies focusing on adults [11, 13, 14], clinical groups [101, 102] or children and/or adolescents [10, 12]. Most studies linking P300 responses to behavioral measures, such as reaction times or working memory capacity, are measured in active conditions and focused on P3b response. As such, they tend to tell us more about task-related responses and reaction times than about more general links between the magnitude or speed of brain processing and behavioral skills. Fuchigami et al. [103] investigated 4 to 21-year-old participants and found that both P3b latency and the reaction time (button push for the target tone) decreased with age. Furthermore, a link between the latency of P3b response in an oddball paradigm and children’s working memory scores (as measured with digit span) has been found in some studies for school-aged children [104–106, both children and adults: 107]. Boucher et al. [104] found also that the latency of the separately measured P3b responses correlated positively with reaction times in a computerized test measuring inhibition, and the P3b amplitudes were negatively associated with the completion times in a section of the Stroop test measuring inhibition. Regarding P3b amplitudes, Barceló et al. [14] found a negative correlation between the P3b amplitudes to task-related sound and the reaction time in the set-shifting task. However, no such correlation was found between the reaction time and a P3b to a novel sound, unrelated to the task. In their study, they further suggested that the processing of novel distracter and familiar task switching have a common neural substrate because the P3 components show similar scalp topographies [14].

Even though P3a response is also thought to be connected with attentional allocation, a lot less seems to be known about its associations with behavioral measures of executive functions. Polich et al. [107] investigated children and adults and found that there was a link between P3a latencies and working memory scores, such that the shorter the latency, the better scores were obtained in a digit span test. Studying the impact that inhibition of distractor-based interference had on visual attention in adults, Cowley [108] showed that intra-modal distractors (primers incongruent to following targets) had much larger effect on P3a amplitude than the task-relevant difference between target types, and this was mirrored by reaction times and error rates. In other words, the requirement to inhibit (incongruent distractors) caused much larger P3a and concomitant slow reaction times, compared to the requirement to shift task (select target types). Furthermore, Saarikivi et al. [10] found that high-performing adolescents in a set-shifting task (inhibition subtest in Nepsy II, [109]) showed larger P3a amplitudes for musically relevant deviant stimuli than their low-performing peers. This difference was seen in a group of 13–15-year-old adolescents but not in the younger group, consisting of 9–11-year-olds. To summarize, the first study indicates a link between working memory scores and P3a latency, and the second proposes that the inhibition and set-shifting tasks affect differentially to the related P3a amplitude. The third study by Saarikivi et al. [10] suggests that P3a amplitude may be associated with performance in set-shifting task. Regarding our study, this is the most relevant result as it focuses on auditory responses and reaction times connected to set-shifting.

### The present study

Our cross-sectional study focused on associations between the P3a responses and inhibition and set-shifting skills in 10-year-old children. To investigate our hypotheses, we conducted measurements with forty-two 10-year-old children. As our P3a study paradigm, we used well-established multi-feature paradigm [83, 110, 111]. It consists of one standard stimulus and several deviant and novel stimuli and was chosen because we did not have a justifiable hypothesis for choosing a specific acoustic feature in the current context but, instead, were probing the associations between neural and behavioral indices of development of executive functions at more general level. In the P3a study paradigm, non-standard stimuli are presented in an alternating order with the standard and they consist of four different deviant sounds and novel sounds. Each deviant stimulus differs from the standard stimulus in one feature (frequency, duration, location, gap). Novel stimuli (35 different ones) are machine sounds from everyday life, such as car honk, doorbell, or telephone ringing. This paradigm enables fast data collection at once for both acoustically small deviances and largely different novel sounds. The executive functions of the children were probed by a modified flanker task. It enables one to determine their reaction times reflecting their performance regarding inhibition and set-shifting skills.

During childhood maturation, the P3a amplitudes seem to have two parallel trajectories depending on the auditory paradigms: it has been found to increase for small deviants from the standard stimulus and decrease for novel sounds, while reaction times in inhibition and set-shifting paradigms become faster [37, 48, 110–112]. However, there is no evidence about these phenomena from one experimental study. Thus, our first hypothesis H1 is: The faster inhibition and set-shifting processing is connected to larger P3a amplitudes for deviants and smaller P3a amplitudes for novel stimuli. Furthermore, as both the reaction times reflecting inhibition and set-shifting and P3a latencies tend to decrease during childhood, our second hypothesis H2 is: The faster inhibition and set-shifting processing is connected to shorter P3a latencies for both deviants and novel stimuli.

To investigate our hypotheses, we conducted linear mixed-model analyses, in which the P3a amplitudes and latencies for deviants (pooled over all deviant stimuli) and novel sounds acted as dependent variables and were predicted by reaction times reflecting inhibition and set-shifting. The children’s language and special needs education statuses were also included in the model as predictors, as it was deemed possible that they modulate P3a responses.

## Results

Table 1 lists the reaction times (mean and SD) as well as performance accuracy of all the blocks of the modified flanker task.

**Table 1:** Mean reaction times and standard deviation (SD) in milliseconds for accurate responses and the percentage of accurately responded trials for all the separate task types in all blocks

	Reaction times (ms)		Accuracy (percentage)		
	Mean (SD)	[min; max]	Accepted (SD)	Median	[min; max]
**Block 1** (all congruent)	458 (63)	[316; 632]	97.6 (3.7)	100.0	[85.0; 100.0]
**Block 2**					
Congruent	482 (67)	[345; 629]	99.3 (2.1)	100.0	[90.0; 100.0]
Incongruent	523 (75)	[358; 657]	95.8 (5.3)	95.0	[75.0; 100.0]
Mean Block 2	503 (68)	[358; 619]	97.6 (0.0)	97.5	[85.0; 100.0]
*RT*_*INH*_: *Block 2*_*Mean*_ − *Block 1*_*Mean*_	45 (48)	[− 40^a^; 144]	N/A	N/A	N/A
**Block 3**					
No switch, congruent	773 (149)	[548; 1237]	98.3 (6.6)	100.0	[60.0; 100.0]
No switch, incongruent	999 (198)	[650; 1386]	89.5 (11.9)	90.0	[60.0; 100.0]
Switch, congruent	813 (178)	[534; 1392]	97.6 (9.6)	100.0	[40.0; 100.0]
Switch, incongruent	1020 (189)	[657; 1484]	88.6 (12.6)	90.0	[50.0; 100.0]
Mean Block 3	901 (152)	[614; 1326]	93.5 (0.1)	95.0	[68.0; 100.0]
*RT*_*SS*_: *Block 3*_*Mean*_ − *Block 2*_*Mean*_	398 (127)	[122; 714]	N/A	N/A	N/A

RT_SS_: reaction time for set-shifting; RT_INH_: reaction time for inhibition

^a^Six children were faster in tasks measuring inhibition than in the congruent task, resulting in a negative value for minimum RT

**Fig. 1:**
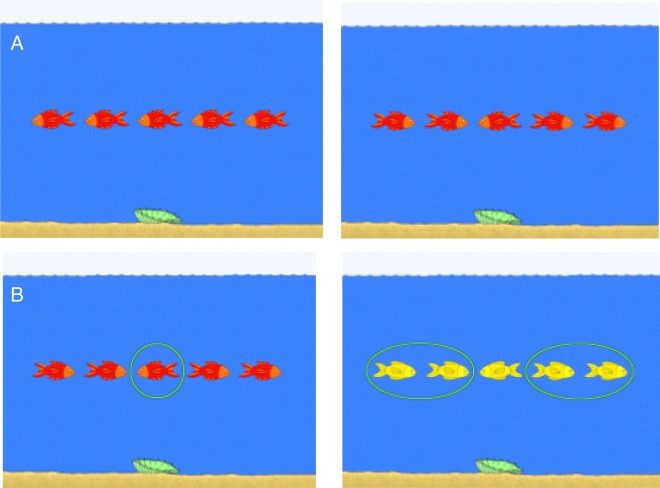
Blocks 2 and 3 in the flanker task. **A** (top) represents the congruent (left) and incongruent (right) trials in Block 2, in which the child should push the button according to the direction of the middle fish. **B** (bottom) represents the trials in Block 3, in which the target fish varies according to the color of the fish. When the fish are red (left) the child should push the button according to the direction of the middle fish, and when the fish are yellow (right) the child should push the button according to the direction of the flanker fish. Block 3 includes both congruent and incongruent trials

**Fig. 2:**
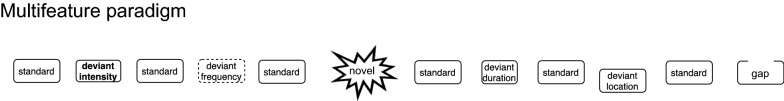
A multifeature paradigm. Standard stimuli were intervened by deviant sounds (intensity, frequency, duration, location, gap) and different novel sounds. This sound sequence was presented via headphones for 10 min and 30 s while the participants were instructed to watch a silent video and to ignore the sounds

The reaction times reflecting inhibition and set-shifting did not correlate (p = 0.772). Neither did the mean amplitude and latency of the P3a response correlate, as determined separately for each deviant and novel sound (p = 0.108–0.852). Figure 1 displays the modified flanker task and Fig. 2 the multifeatured task used to measure P3a.

**Fig. 3:**
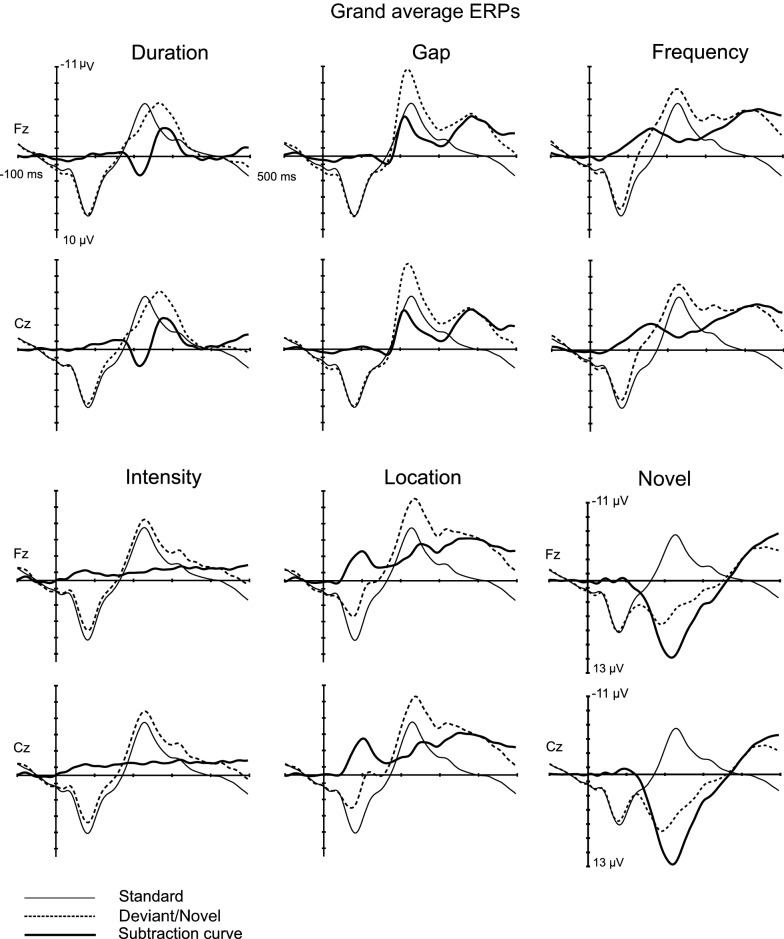
Grand average standard and deviant responses along with subtraction curves for deviant and novel stimuli averaged over all participants on electrodes Fz and Cz. Note that in the analyses the defined latencies and amplitudes for duration, gap, frequency, and location were pooled. As the response for intensity deviant did not show any sign of either MMN or P3a components, it was left out of the subsequent analyses

**Fig. 4:**
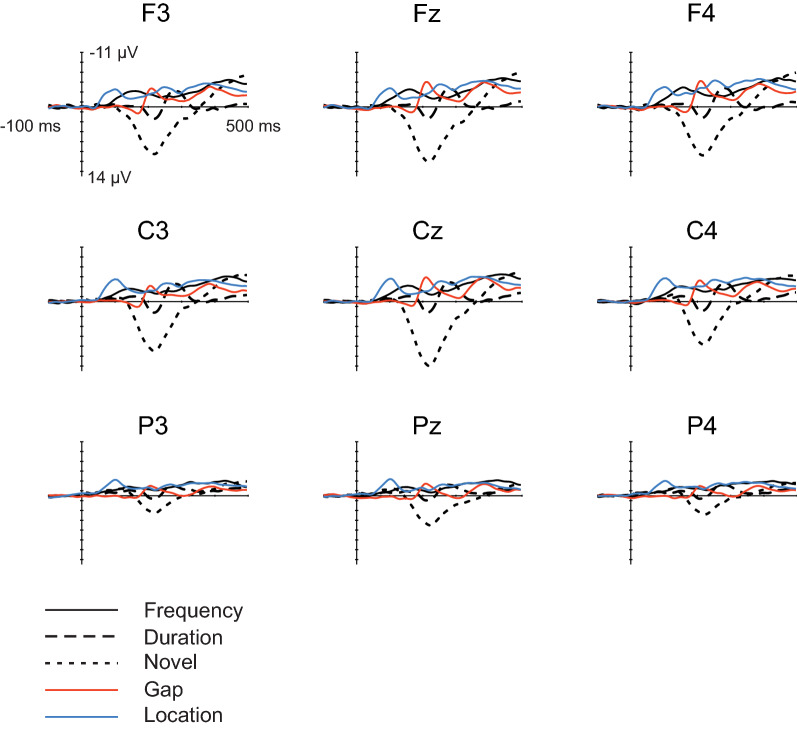
Subtraction waveforms for all inspected deviants and novel sound. Note that in the analyses the defined latencies and amplitudes for duration, gap, frequency, and location were pooled. As the response for intensity deviant did not show any sign of either MMN or P3a components, it was left out of the subsequent analyses

Figure 3 depicts responses to standard and deviant sounds along with subtraction curves for deviants and novel stimulus averaged over all participants on electrodes Fz and Cz and Fig. 4 displays the inspected subtraction waveforms on electrodes F3, Fz, F4, C3, Cz, C4, P3, Pz, and P4. Table 2 lists the number of the trials included in the analyses and Table 3 lists the mean, SD, and median values for the peak latencies and mean amplitudes of P3a.

**Table 2:** The trials per stimulus type included in the analysis

	Accepted trials (%)			
	Mean (SD)	Median	Min	Max
Standard	98.0 (5.5)	99.3	64.3	100.0
Duration	98.2 (4.6)	100.0	71.0	100.0
Gap	98.5 (4.9)	99.5	68.0	100.0
Frequency	98.3 (5.1)	100.0	68.0	100.0
Location	98.3 (4.6)	100.0	71.1	100.0
Novel	97.8 (6.6)	99.0	57.0	100.0

The total number or trials was 630 for the standard stimuli and 105 for each non-standard stimuli

**Table 3:** The descriptive statistics of P3a response for the individual peak latencies and individual mean amplitudes (50 ms around the peak amplitude)

	Peak latency (ms)			Mean amplitude (μV)		
	Mean (SD)	Median	[min; max]	Mean (SD)	Median	[min; max]
Duration	379^a^ (39)	378	[312; 446]	0.92 (3.63)	0.30	[− 5.96; 10.01]
Gap	297^a^ (29)	295	[238; 370]	− 0.31 (3.82)	− 0.67	[− 8.88; 6.90]
Frequency	252 (43)	251	[184; 376]	− 0.89 (3.36)	− 0.61	[− 8.46; 6.64]
Location	277 (42)	281	[214; 362]	− 2.24 (3.44)	− 1.98	[− 8.32; 4.81]
Novel	217 (17)	218	[182; 276]	11.61 (4.76)	11.43	[2.43; 20.21]

Both the latencies and the amplitudes are averaged over Fz and Cz electrodes

^a^Notice that the onset of deviation is 65 ms later for duration deviation and 42 ms later for gap deviation compared to other non-standard stimuli

According to linear mixed model, children’s language or special needs education status did not have a main effect on the P3a amplitudes for deviant stimuli [*F*(1, 36) = 1.290, *p* = 0.264; *F*(1, 36) = 0.197, *p* = 0.660, respectively]. Furthermore, neither the RT_INH_ nor RT_SS_ had a main effect on the P3a amplitudes [*F*(1, 36) = 0.798, *p* = 0.378; *F*(1, 36) = 0.002, *p* = 0.969, respectively]. This indicates that reaction times reflecting inhibition or set-shifting were not connected to the magnitude of averaged P3a amplitude for deviant stimuli. No significant interaction was found between the children’s language and special needs education status (*p* = 0.682).

The children’s language or special needs education status did not have a main effect on the P3a latencies for deviant stimuli [*F*(1, 36) = 0.002, *p* = 0.962; *F*(1, 36) = 0.033, *p* = 0.857, respectively]. The reaction time for inhibition had a significant main effect on the P3a latency for deviants [*F*(1, 36) = 4.532, *p* = 0.040], whereas the reaction time for set-shifting had no such effect [*F*(1, 36) = 0.040, *p* = 0.843]. Estimated marginal means evaluating the direction of the association between the P3a latency and *RT*_*INH*_ showed that the first and third quartiles of *RT*_*INH*_ (Q1 = 14 ms and Q2 = 81 ms) corresponded with P3a latencies of 296 ms and 307 ms. This indicates shorter response latency for children showing faster reaction times related to inhibitory skills. No significant interaction was found between the children’s language and special needs education status (*p* = 0.115).

Conducted linear mixed models revealed no significant main effect of children’s language status on P3a amplitudes or latencies for novel stimuli [*F*(1, 36) = 0.530, *p* = 0.471; *F*(1, 36) = 0.874, *p* = 0.356, respectively]. Instead, the special needs education status did have a main effect on both P3a amplitudes and latencies [*F*(1, 36) = 5.176, *p* = 0.029; *F*(1, 36) = 4.899, *p* = 0.033, respectively]. There was no significant main effect of RT_INH_ or RT_SS_ on the P3a amplitude for novel stimulus [*F*(1, 36) = 0.002, *p* = 0.964; *F*(1, 36) = 0.074, *p* = 0.788, respectively]. Similarly, no significant main effect of neither RT_INH_ nor RT_SS_ on the latency of the novel P3a was found [*F*(1, 36) = 0.031, *p* = 0.861; *F*(1, 36) = 2.173, *p* = 0.149, respectively]. No significant interaction was found between the children’s language and special needs education status for either amplitude or latency *p* = 0.831, *p* = 0.624, respectively).

**Fig. 5:**
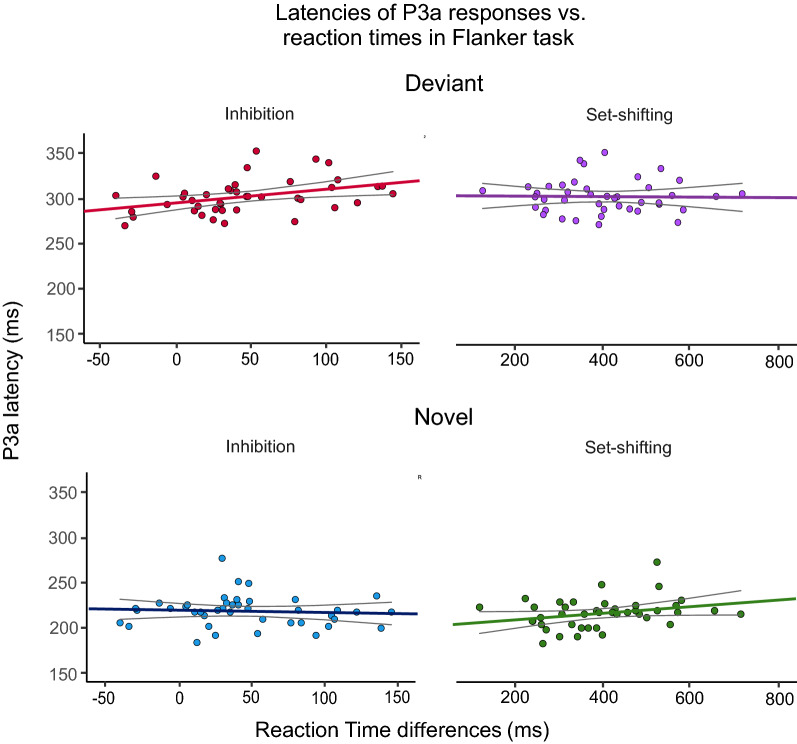
Individual P3a latencies for deviant and novel stimuli along with reaction times related to inhibition and set-shifting in the modified flanker task. The colored lines represent ordinary least square regression lines and the areas between the grey lines show 95% confidence interval for standard error of the mean

Figure 5 depicts the relationship between the individual P3a latencies, and the reaction times related to inhibition and set-shifting.

## Discussion

The aim of our exploratory study was to investigate the associations between the executive functions and P3a response in 10-year-old children. More specifically, we wanted to know if the reaction times related to inhibitory skills and set-shifting in a modified flanker task are linked to the magnitude (Hypothesis 1) and latency (Hypothesis 2) of the P3a for small auditory deviants and novel stimuli in a separate multifeature ERP paradigm.

Our results confirmed H2 partly: there was a significant association between P3a latency and separately measured reaction time in the flanker task: faster reaction times related to inhibitory control were connected to shorter P3a latencies for deviants. H1 was not confirmed: no associations were found between P3a amplitudes and the response speed related to inhibitory skills or set-shifting. Former studies have not reported such connection between P3a latency and reaction times related to inhibition, but it is impossible to know whether this is due to the lack of research or non-reported (null) results.

The previous correlational studies have shown that reaction times for tasks measuring inhibition and set-shifting tend to be shorter in younger compared to older children at least before late teenage [50, 112, 113]. Also, the P3a latencies for both novel and deviant sounds seem to decrease with age in childhood [100]. If both latencies were connected to both reaction times, one could infer that these associations result from individual processing speed affecting all reaction times and response latencies in children. However, our results point to a more specific association between reaction times related to inhibition and P3a latency for deviant sounds. This suggests that shared neural processes underlie these two mechanisms.

Contradicting our hypothesis, the P3a amplitudes for small auditory deviants, as well as for novel sounds, were not associated with reaction times related to either inhibition or set-shifting. Saarikivi et al. [10] found a link between the performance in a set-shifting task and P3a amplitudes in a subpopulation of their study. They studied 9–11 and 13–15-year-old children and found that only in the group of adolescents, the larger P3a amplitude was associated with higher performance (i.e., faster completion times) in set-shifting task. As we did not find any link between the P3a amplitude and set-shifting skills in 10-year-old children, our study is in line with the results regarding the younger age-group. Inhibition and set-shifting abilities seem to be still developing at the age of 10 [39, 50, 58–60, 112–114], but the research on the developmental P3a amplitude has been scarce, and the maturational peak of the P3a amplitudes is not clear [100]. Regarding P3b, van Dinteren et al. [115] concluded in their review that the P3b amplitudes reached their peak before the response latency reached its minimum during adolescence. They further discuss the possibility that whereas response latency (as well as reaction times recorded in the same paradigms) may be linked to overall neural speed e.g., due to development of myelination, the amplitude changes reflect different brain processes, e.g., the strength of cognitive response. Even though the P3a seems to (at least partly) reflect other cognitive processes than the P3b, the latency and amplitude might be linked to neural processes in similarly divergent patterns.

Our study design aimed to avoid the task-impurity problem by using the differences in reaction times as markers for separate EF skills. RT_INH_ and RT_SS_ did not correlate with each other within our sample. In addition, only the reaction time related to inhibition was associated with the P3a latency (for deviance). One could speculate that this is due to inhibitory and set-shifting skills having diverged from one another by the age of ten, but our results as such, do not prove this to be the case.

There seems to be a lack of research on the maturation of both EF skills and P3a responses, along with the connections between them throughout late childhood and adolescence. To fill this gap, longitudinal studies covering several years, with substantial sample sizes would be optimal. With such studies it would be possible to assess the developmental trajectories of these features, and thus gain more knowledge about the properties that have an impact on everyday life of adolescents and young adults.

There are some shortcomings in the present study. The sample size of the study was not very large, and this diminishes the impact of the results. Unfortunately, recruiting participants within one school limits the number of possible recruits within one age group. Furthermore, in our case, several children refused to participate in EEG measurements even if they and their guardians agreed to participate in behavioral part of the study, thus decreasing the number of children with both flanker test and EEG data.

The high number of children with special needs education in our study may raise some questions of the sample’s representativeness. Even if this had not been taken this into account in the analyses, it would not have compromised the results in our opinion. The sample did not include children with severe developmental disabilities, and all children participated general education in which—following Finnish tiered frame for special needs education—intensified support is given by special educators if general support is insufficient. Furthermore, some original participants with outlier values for reaction times and latencies were excluded from the analyses. If all children receiving special needs education would be excluded from studies, the sample would represent more high-functioning population (cf. psychology students’ cognitive abilities) and less middle- to lower-middle class population. Due to the research being conducted in the Finnish school, which is inclusive, the participants represent more heterogenous sample than is possible to achieve with a study where measurements are conducted in laboratory settings. Parents who have resources to bring their children to these settings, represent typically higher socioeconomic class, and this diminishes the validity of many results obtained via such data collection.

Having longer inter-stimulus intervals in the ERP paradigm might have resulted in more salient P3a components (of positive polarity) for the deviations. Now the response for the following stimulus supposedly overlaps with the previous response, thus interfering the investigation of the phenomena. The future studies should acknowledge this shortcoming in their design. Of note is also that although we tried to avoid the task-impurity problem by using reaction time differences, it is probably not possible to totally separate inhibition and set-shifting skills from each other in task performance. The task demands also change from one block to another and the increased reaction times may at least partly be due also to other increased demands than those emerging from inhibition and set-shifting.

Finally, it is important to acknowledge that even though the present study brings information about the developmental stage regarding 10-year-old children’s executive functions and brain measures, it is not genuinely a developmental study. Such study would need either a longitudinal design or at minimum a cross-sectional comparison of different age-groups.

## Conclusions

To our knowledge, this is the first time that a link between the latencies of separately measured P3a responses and behavioral measures for inhibition has been found, particularly in school-aged children. Our research revealed that unlike the P3a amplitudes, the P3a latency for deviant stimuli are linked to the reaction times reflecting inhibitory skills. These results are in line with the view that inhibition and P3a latency are linked via shared neural mechanism. Thus—on group level—it appears that the same factors that contribute to the speed of the early sensory level attentional processing regarding small and possibly irrelevant stimuli (as indexed by P3a), also contribute to the speed with which the irrelevant stimuli are inhibited in a behavioral task.

## Methods

The data were collected as part of a larger ArtsEqual project (www.artsequal.fi; Academy of Finland https://www.aka.fi/en/strategic-research/) investigating the effects of music and movement interventions on children’s cognitive, academic, and social skills and motivation. The participants were recruited from four parallel classes in one municipal school representing a lower-middle class area within the Helsinki metropolitan region. The guardians signed the informed consent, and the children were asked for their verbal assent before the experiment. The data used in the present study were collected between October and December 2017. This was the first EEG measurement, and the fourth flanker task testing the children participated in during the whole project (September 2016–May 2018).

### Participants

Out of the original 73 children participating in the larger study, 46 participated in both the flanker task and the passive oddball. Three participants were further excluded from the analyses due to showing difference reaction times (RT) that deviate more than three standard deviations (SD) from the mean RTs and one participant due to showing a P3a latency (for novel sound) more than three SDs longer than the mean latency. As a result, 42 children (19 female; 31 monolingual Finnish speakers; 15 children receiving special needs education^1^), who participated in both measurements, were included in the sample. The children were 9–10-year-old 4th graders (mean age when attending the EF test 10 years, 3.9 months, SD 3.8 months; mean age when attending the EEG-measurement 10 years, 5.2 months, SD 3.8 months).

### Procedure

The flanker tasks and the EEG measurements were conducted on separate days at the children’s school, in a room with only the child and the experimenter(s) present. The behavioral flanker task took approximately 20 min. During the EEG measurement participants watched a muted movie with subtitles and listened to the stimuli via headphones (Sony Professional MDR-7506). They were instructed not to pay attention to the sounds and sit as still as possible. The measurement with preparations took approximately 1.5 h out of which the paradigm reported here took ten minutes and was conducted right after the preparations. The children were offered snacks during the breaks and a sticker as a reward.

### The ERP paradigm

A multifeature paradigm [116], presenting a standard sound and six different sound categories, was used in the present study. Typically, in the multifeature paradigm, every other sound is a standard sound, and is always followed by a deviant sound (Fig. 2). Each deviant sound differs from the standard sound only in one feature, and thus, the change in one specific sound feature happens only in approximately 5–15% of the stimuli, depending on the paradigm. This method enables a fast means to collect data, which is an important feature especially when working with children who typically find it hard to sit immobile for long periods of time. In the present study, novel stimuli differing considerably from the standard and deviant stimuli was also used. In earlier studies, responses have been found to be similar in oddball and multifeature paradigms, for both children and adults [110, 117–119].

In addition to standard stimulus, the paradigm consisted of novel sounds and deviant stimuli that differed from the standard tone in only one feature, either in sound duration (DUR), frequency (FRE), intensity (INT), location (LOC) or having a gap (GAP) in the middle of the sound. Each deviant sound and novel sounds appeared in approximately 8.3% of the stimuli.

The standard stimulus was 100 ms in duration (including 10 ms rise and fall times) and was composed of three sinusoidal partials, namely 500, 1000, and 1500 Hz because such harmonically rich sounds evoke larger MMN and P3a responses than pure sinusoidal tones [120, 121]. The intensities of the second and third partials were lower than that of the first partial by 6 dB and 10 dB, respectively. There were two types of stimuli for each of the frequency and location deviants. A half of the frequency deviants were 10% higher (partials: 550, 1100, 1650 Hz) and the other half 10% lower (450, 900, 1350 Hz) than the standard. A change in the perceived sound-source location was created evenly to the left and to the right channels with an interaural time difference of 900 μs and an average intensity difference of 4 dB between the channels, the louder channel representing the source of the sound. Thus, the perceived difference between the standard stimulus and the location deviant was ∼ 90°. The intensity deviant was − 5 dB compared with the standard, and the duration deviant was 35 ms shorter than the standard, i.e., 65 ms in duration. Further, the gap deviant was constructed by cutting out 16 ms (6 ms fall and rise times included) from the middle of the standard stimulus, leaving there a silent gap. To sustain the novelty of a novel stimulus, this stimulus category was comprised of 35 different machine or other artificial non-human or non-animal sounds, differing substantially from the standard tones. Each separate novel sound was presented altogether three times.

The stimuli were presented via Sony Professional MDR-7506 headphones at an average of 70 dB with equal phase and intensity at both ears, excluding location deviant. The stimuli were presented with Presentation 20.0 (Neurobehavioral Systems, Inc., Albany, CA, United States), the interval between the onsets of consecutive sounds being 500 ms. The paradigm lasted for 10 min and 30 s and included 1260 stimuli, out of which 50% were standard sounds.

### The modified flanker task

In the current study, the modified flanker task was used to measure the inhibition and set-shifting. The flanker task is based on the assumption that reacting to the target stimulus is distracted by the surrounding stimuli (flankers), especially if they contradict the response to the task—e.g., point in a different direction or present a different object. This distraction is seen in the accuracy of the responses and particularly in the reaction times, which become slower as the cognitive load of the task increases due to inhibitory control. According to previous studies, the RTs (for correct answers) in different flanker versions become faster throughout childhood and adolescence [122, 123] and this change can be thought of as marking the enhancement in the EF related skills.

The original flanker task [29] used target and distraction letters, but later, the flanker task was modified to several versions using e.g., arrows and fish figures pointing in the same and different directions [124–126]. Furthermore, in some studies a section in which the target changes according to rules (mixed flanker task) has been added [126, 127].

In the current study, the participant saw the stimuli comprised of five fish on the computer screen and was instructed “to feed the fish” by pressing the left hand button if the target fish was swimming to the left and the right hand button if the swimming direction was to the right (Fig. 1). The flanker task was presented with a Dell Latitude E7450 computer, the size of the fish was 23.0 × 10.2 mm, and the distance between them was 4.7 mm. The fixation cross was 3.4 × 3.4 mm in size and dark blue (RGB 5, 29, 79) and the background color was lighter blue (RGB 59, 127, 251). The children sat about 50 cm from the screen and gave their responses with a Cedrus RB-730 response box (Cedrus Corporation, San Pedro, CA, USA). In the first block of the modified flanker task, the target fish was always in the middle of the row, and the surrounding four fish (two on each side) swam in the same (congruous) direction (20 trials). In the second block, the target fish was also always in the middle, and swam in the same (congruous) or in the opposite (incongruous) direction (20 trials, each). In the third block, the target stimulus varied: if the fish were red (as they were in the first and second blocks), the target fish was the one in the middle; if the fish were yellow, the task was to feed the surrounding fish who all swam in the same direction. In addition to having congruent and incongruent conditions, the task also included either switching or not-switching from the previous separate task. Thus, the third block included 40 separate trials, switch/congruent (10), switch/incongruent (10), no switch/congruent (10) and no switch/incongruent (10). Therefore, there were 100 trials: within each block, the trials were presented in random order. The blocks were always presented in the same order.

Before each block the children were explained what they were supposed to do: whether to push the button to feed all fish (first block), the middle fish (the second block) or variate between the middle and flanker fish according to the color of the fish (third block). Children rehearsed the task before each block to ensure they understood it. If the child responded inaccurately to at least three of the five rehearsal tasks for inhibition, or to at least four of the eight rehearsal tasks for set-shifting, the instructions and rehearsal were repeated. However, very few participants needed repetition. The test trials were not included in the analyses. Before each task, a fixation cross appeared at the center of the screen for 100 ms. After that, the task appeared on the screen, staying there for 7000 ms if the child did not respond before that. After each block, the child was given positive feedback without revealing the quality of the performance. The flanker task took approximately 20 min, including rehearsals before each block.

Each type of flanker task always taps more than one executive function factor, and it is impossible to disentangle them completely. Due to this task-impurity problem [see e.g. 49], earlier research used different strategies to tap the specific EF skills with flanker tasks. In analyses using children’s reaction times, inhibitory control had been calculated from mean RTs from incongruent and congruent trials [128]; mean RTs from neutral, incongruent and congruent trials [125]; mean RTs from only incongruent trials [18, 124, 129, 130]; or means from the RT difference between incongruent and congruent/neutral trials [26, 126]. There are fewer examples about measuring set-shifting abilities of children with flanker task, but both Röthlisberger et al. [126] studying preschool children, and Li and Dupuis [127] studying young adults, used the mean RTs over congruent and incongruent trials in the shifting (switch) trials as an index for cognitive flexibility.

In the present study, we aimed to resolve the task-impurity problem by using differences between reaction times in the analyses. The measured reaction times for congruent trials in Block 1 (no inhibition task) differ significantly from the RTs for congruent trials in Block 2 [t(41) = − 3.417, p = 0.001].This suggests that the inhibitory control is in use when participants are monitoring the screen in Block 2, both in congruent and incongruent trials. Thus, RT_INH_ reflecting inhibition was calculated by subtracting the RTs of the first block (congruent, all fish swimming in the same direction in every task) from the mean RTs for the second block (congruent and incongruent trials, the surrounding fish swimming in the same or the opposite direction as the middle, target fish). Similarly, as the measured reaction times for congruent and incongruent no-switch trials in Block 3 did differ statistically significantly from the congruent and incongruent trials in Block 2 measuring inhibition [congruent trials: t(41) = 14.549, p < 0.001; incongruent trials: t(41) = − 17.350, p < 0.001], it appears that the set-shifting cost is also present in the trials in which no switch happens but the participant is prepared for the set-shifting in the task. Thus, RT_SS_ reflecting set-shifting was calculated by subtracting the mean RTs of the second block (congruent and incongruent, target fish always in the middle) from the mean RTs over all trials in the third block (switch/congruent, no-switch/congruent, switch/incongruent, and no-switch/incongruent; target fish either the middle one or the surrounding ones). See Table 1 for all the reaction times.

As the hit rate for the responses was very high [mean accuracy = 95.9%, median = 97.0%, SD = 3.6%], we did not use the number of accurate/inaccurate responses in the analyses. Only the reaction times for correct responses were included in the analyses. See Table 1 for the descriptive statistics of accuracy in flanker task.

### Data recording and processing

A portable EEG equipment (BrainVision LiveAmp amplifier and BrainVision Recorder; Brain Products GmbH, Gilching, Germany) was used in the measurements. The EEG was recorded with 32 Ag–AgCl scalp electrodes postitioned according to the international 10–20 system by using ActiCap (Brain Products, Germany). The EEG data were registered with sample rate of 500 Hz. Recording reference was Fpz or FCz depending on the size of the used cap. Out of the 32 active electrodes of the cap, two were placed on the mastoid bones and one below the left eye. The recorded data were processed with CTAP software (The Computational Testing for Automated Preprocessing toolbox, https://version.helsinki.fi/hipercog/ctap; [131, 132]). Highpass was filtered at 0.5 Hz (length 3381 points), followed by lowpass filtering at 30 Hz (length 227 points), using a Hamming windowed sinc finite impulse response (FIR) filter provided by the pop_eegfiltnew function from Andreas Widmann's firfilt toolbox (https://github.com/widmann/firfilt). Independent Components Analysis (ICA) was computed, and artefactual ICs were identified via statistical methods from CTAP, focused on spectral and ocular artefacts [14% of all ICs (SD 5%)]. After this, noisy electrodes [average 3.1 (SD 1.8) electrodes] were detected in CTAP by using channel-wise statistics from the FASTER toolbox [133]; as described in Cowley et al. [131], and replaced by spherical interpolation from surrounding channels. For the analyses, the data were re-referenced to the mean over mastoid electrodes. Further, the epochs from − 100 ms before to 600 ms after the stimulus onset were extracted from the data, excluding those epochs where amplitudes exceeded ± 120 μV [0.5% of all epochs (SD 0.8%)]. The responses for each stimulus type were averaged for each participant and exported to MATLAB 2016a (The MathWorks Inc., Natick, MA, United States).

To follow the analysis path of the previous papers using the multifeature paradigm [83, 110, 111], the responses for both higher and lower frequency deviants were combined, as well as location deviants for tones coming from both left and right direction. Averaged standard responses were then subtracted from the averaged deviant/novel responses, separately for each participant and these subtraction curves were used in the analyses. This method has been used in several studies [e.g., 6, 134–137], and is an established method especially regarding multifeatured paradigms with children as participants [84, 110, 111]. After preliminary analyses showing no lateralization effects, the mean responses over Fz and Cz electrodes were chosen for the final analyses, as the response components show larger amplitudes on the frontal and central lines compared to the parietal line (see Fig. 4). The individual peak amplitudes and their latencies were assessed separately for both electrodes with in-house Matlab toolbox (CBRUplugin, Cognitive Brain Research Unit, University of Helsinki) within the visually defined time windows (plots of mean subtraction curves for each individual) that ranged from 130 to 470 ms from the sound onset. The peak latency was detected as a zero point of first derivative. In cases when several peaks instead of only one were detected in the time window, we selected the one with maximum (or minimum according to the positivity/negativity) amplitude. Individual mean amplitudes over 50 ms time windows were calculated then around the individual peak latencies, separately for each deviant and novel subtraction waveform. Although the onset of deviation is later than the sound onset in duration and gap stimuli, we used the same onset point for all deviants. As the focus of the study is on the associations between P3a latencies and reaction times, subtracting the same number of milliseconds (e.g., 65 ms in duration deviant) from all participants’ peak latencies would not change the relationship between the inspected variables.

The developing P3a responses of children do not reach positive polarity but may show only a notch reaching towards positivity—at least in paradigms with fast stimulation rate [see e.g. 83, 84, 111]. However, as the response for intensity deviant did not show any sign of either MMN or P3a components (see Fig. 3), it was left out of the subsequent analyses. As there was no hypothesis regarding the different deviant sounds, we pooled the stimuli by averaging amplitudes and latencies over duration, frequency, location, and gap stimuli.

### Statistical analyses

Linear mixed-model analyses with restricted maximum likelihood were conducted for amplitudes and latencies for deviants (pooled over DUR, FRE, LOC and GAP stimuli) and novel sounds. Bayesian information criteria was used to define model fit. In all models, RT_INH_, RT_SS,_ children’s language status (native/non-native) and special needs education status acted as independent predictors. Participants were treated as random factors with random intercept. Alpha level was set at ɑ = 0.05.

## Data Availability

The datasets generated and analyzed during the current study are not publicly available due to the small sample size which might enable the disclosure of sensitive information about individual children, but are available from the corresponding author on reasonable request.

## Footnotes

1 The high proportion of multilingual children and children receiving special needs education describes the diversity of the population in the lower-middle class area of the school and importantly, the Helsinki metropolitan area. In Finland, inclusive education is widely used and emphasized. Legislation directs the Finnish education system to be based on equality, justice of learning and on the principle of inclusion.
